# Correction to: J Cardiovasc Magn Reson, volume 24

**DOI:** 10.1186/s12968-022-00847-3

**Published:** 2022-03-16

**Authors:** Chiara Bucciarelli-Ducci

**Affiliations:** Royal Brompton and Harefield Hospitals, Guys’ and St Thomas NHS Hospitals and School of Biomedical Engineering & Imaging Sciences, King’s College London, London, UK

## Correction to: Journal of Cardiovascular Magnetic Resonance, volume 24 https://doi.org/10.1186/s12968-021-00833-1, https://doi.org/10.1186/s12968-021-00835-z, https://doi.org/10.1186/s12968-021-00836-y

In volume 24 of the *Journal of Cardiovascular Magnetic Resonance* there were 3 articles [1–3] with an incorrect affiliation for Chiara Bucciarelli-Ducci. The 3 incorrect and correct affiliations are listed below:

**Table:** 

Article	Incorrect	Correct
https://jcmr-online.biomedcentral.com/articles/10.1186/s12968-021-00833-1	Bristol Heart Institute, Bristol National Institute of Health Research (NIHR) Biomedical Research Centre, University Hospitals Bristol NHS Trust and University of Bristol, Bristol, UK	Royal Brompton and Harefield Hospitals, Guys’ and St Thomas NHS Hospitals and School of Biomedical Engineering & Imaging Sciences, Faculty of Life Sciences and Medicine, King’s College London, London, UK
https://jcmr-online.biomedcentral.com/articles/10.1186/s12968-021-00836-y	Department of Cardiology, Imperial College, London, UK	Royal Brompton and Harefield Hospitals, Guys’ and St Thomas NHS Hospitals and School of Biomedical Engineering & Imaging Sciences, Faculty of Life Sciences and Medicine, King’s College London, London, UK
https://jcmr-online.biomedcentral.com/articles/10.1186/s12968-021-00835-z	Royal Brompton Hospital, London, UK	Royal Brompton and Harefield Hospitals, Guys’ and St Thomas NHS Hospitals and School of Biomedical Engineering & Imaging Sciences, Faculty of Life Sciences and Medicine, King’s College London, London, UK

The original articles have been updated.
